# A Case of Thermal Burns of the Larynx in a Toddler

**DOI:** 10.7759/cureus.75240

**Published:** 2024-12-06

**Authors:** Shinji Iwata, Naoya Nishida, Seitaro Murakawa, Naohito Hato

**Affiliations:** 1 Otolaryngology, Head and Neck Surgery, Ehime University Graduate School of Medicine, Toon, JPN; 2 Otolaryngology, Ehime Prefectural Niihama Hospital, Niihama, JPN

**Keywords:** foreign body aspiration, larynx, microwave oven, stridor, thermal burns

## Abstract

Thermal burns of the larynx are uncommon but can lead to serious upper airway obstruction due to edema and bleeding, especially in children who may struggle to communicate their symptoms effectively. This report presents the case of a one-year-and-seven-month-old boy who developed stridor and respiratory distress after eating a heated potato, which ultimately required tracheal intubation. The initial evaluation suggested foreign body aspiration; however, laryngoscopy confirmed significant arytenoid swelling and airway narrowing due to thermal burns. Following airway management and intensive care with antibiotics and steroids, the patient was extubated on the third day and discharged without complications on the tenth day. This case highlights the importance of considering thermal burns of the larynx in the differential diagnosis of respiratory symptoms after the ingestion of hot food, particularly in young children. Multidisciplinary collaboration between otolaryngologists, pediatricians, and anesthesiologists is essential for the optimal management of such cases.

## Introduction

Thermal burns of the larynx are rare, although thermal burns in the oral cavity and pharynx are possible [1-3]. It is a dangerous condition that can cause upper airway stenosis or obstruction due to edema and bleeding, potentially leading to asphyxiation [4,5]. The symptoms resemble foreign body aspiration or severe upper respiratory tract infections, making history-taking and laryngeal examination crucial [6]. However, diagnosis can be challenging in toddlers, as they may not effectively communicate their complaints and challenges in examining their upper airways. This report presents a case of thermal burns of the larynx in a toddler who required tracheal intubation.

## Case presentation

**Figure 1:**
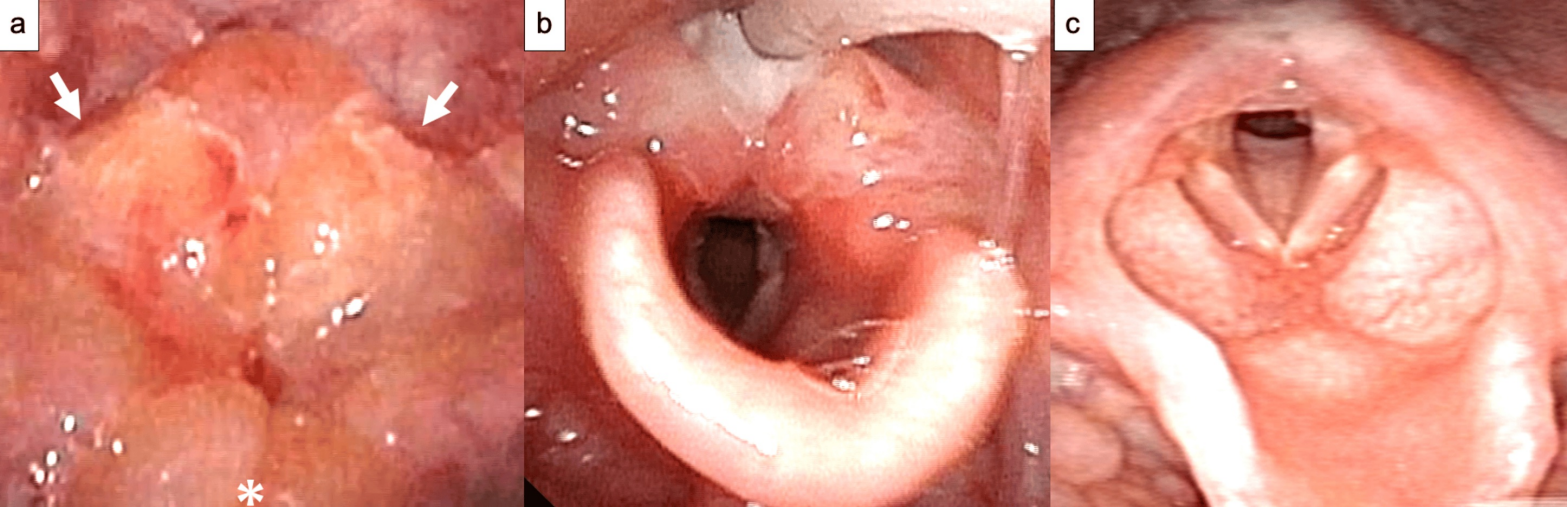
Laryngoscopy (a) The arytenoid area (white arrow) and epiglottis (asterisk) were significantly swollen (5 hours after onset); (b) although redness and edematous swelling of the arytenoid area persisted, the vocal cords were sufficiently visible (3 days after onset); (c) a spot of white mucosal changes were present in the arytenoid area, but the edema had resolved (10 days after onset).

A previously healthy one-year-and-seven-month-old boy started crying and became irritable after eating a heated taro potato for dinner. The crying persisted, and stridor developed, prompting the parents to bring him to our emergency department five hours after onset. His vital signs were as follows: body temperature, 36.7 °C; blood pressure, 148/91 mmHg; heart rate, 186 beats/min; respiratory rate, 18 breaths/min; and oxygen saturation, 97% (ambient air). The skin showed no rashes, and the oral cavity showed no burns. Table 1 displays the results of the blood tests conducted during the initial presentation. Suspecting foreign body aspiration based on the patient’s history, we performed neck and chest radiography; however, there were no abnormalities in the trachea or lungs. For upper airway evaluation, an otolaryngologist performed a laryngoscopy, which revealed significant swelling in the arytenoid area and airway narrowing (Figure 1a). After consultation with pediatricians, otolaryngologists, and anesthesiologists, airway management was deemed necessary. A tracheotomy set was prepared in case of difficulties with oral intubation. An anesthesiologist successfully performed oral intubation using video laryngoscopy, and the patient was admitted to the intensive care unit. Antibiotics (sulbactam/ampicillin, 160 mg/kg/day) and steroids (dexamethasone 3.3 mg/day) were administered intravenously. Daily laryngeal endoscopy was performed to monitor the improvement in airway narrowing, and extubation was performed on the third day post-injury. Although the redness and edematous swelling of the arytenoid area persisted, the vocal cords were visible (Figure 1b). Oral intake was resumed on the sixth day, and the patient was discharged home on the 10th day without fever or respiratory symptoms. Follow-up laryngoscopy at discharge showed white mucosal changes in the arytenoid area; however, the edema resolved (Figure 1c). Informed consent was obtained from the patient's parents for the publication of this case report.

**Table 1:** Laboratory results WBC: white blood cells; RBC: red blood cells; PT: prothrombin time; INR: international normalized ratio; APTT: activated partial thromboplastin time; AST: aspartate transaminase; ALT: alanine transaminase; ALP: alkaline phosphatase.

Test	Results	Reference range
Complete blood count		
WBC	27.6 × 10^3^/μL	3.3–8.6 × 10^3^/μL
RBC	4.28 × 10^3^/μL	4.35–5.55 × 10^3^/μL
Hemoglobin	12.6 g/dL	13.7–16.8 g/dL
Hematocrit	37.6 %	40.7–50.1 %
Platelets	55.7 × 10^4^/μL	15.8–34.8 × 10^4^/μL
Coagulation profile		
PT	120.9 %	70.0–130.0 %
PT(INR)	0.91	
APTT	26.9 seconds	24.0–34.0 seconds
Liver function tests		
AST	36 U/L	3–38 U/L
ALT	17 U/L	4–44 U/L
ALP	227 U/L	38–113 U/L
Total protein	7.3 g/dL	6.6–8.1 g/dL
Albumin	4.8 g/dL	3.7–5.1 g/dL
C-reactive protein	0.021 mg/dL	0.00–0.40 mg/dL
Urea and electrolytes		
Sodium	139 mEq/L	136–148 mEq/L
Potassium	4.5 mEq/L	3.6–5.0 mEq/L
Chloride	106 mEq/L	98–108 mEq/L
Urea nitrogen	14.6 mg/dL	6.0–21.0 mg/dL
Creatinine	0.22 mg/dL	0.65–1.09 mg/dL

## Discussion

Generally, when something hot is placed in the mouth, it reflexively spits out immediately, making thermal burns of the larynx rare [7]. However, thermal burns of the larynx have been reported in children with underdeveloped temperature recognition of food or adults with reduced oral temperature recognition, such as those wearing dentures or inebriated [6,8,9]. Thermal burns of the larynx share clinical features with infectious epiglottitis, and shared symptoms include wheezing, dyspnea, and drooling [10]. In this case, based on the history of onset immediately after eating and normal findings in the oral cavity, we first suspected foreign body aspiration. However, the diagnosis was established after a radiographic examination revealed no foreign bodies, and a final diagnosis of thermal burns of the larynx was made after performing a laryngoscopy.

The food that caused the thermal burns of the larynx was a potato heated in a microwave oven. A microwave oven cooks food using dielectric heating. The dielectric heating mechanism of microwaves results in heterogeneous heating of food, creating uneven heating patterns known as "hot spots” [7]. This allows hot food to bypass the oropharynx and subject the epiglottis and arytenoid area to intense thermal injury [11]. The outer layer may not feel hot; however, as it crumbles during chewing, the hot inner layer can cause laryngeal burns. In this case, the medical history obtained from the parents revealed that the patient had briefly chewed and swallowed food. As a result, the time for which the hot potato remained in the oral cavity was minimal, just enough for the heat to be sensed, leading to almost no thermal injury to the oral mucosa. However, it was hypothesized that burns to the epiglottis and arytenoid area occurred when the potato moved to, and remained in, the pharynx.

It is difficult to accurately predict the onset and severity of airway edema, and little is known about the natural course of airway pathology following burns [12,13]. Depending on the extent and severity of burns, upper airway edema may develop, potentially leading to airway narrowing or obstruction [14]. The peak of laryngeal edema due to thermal burns of the larynx is reported to occur 8-36 hours after injury and may persist for several days depending on the severity [15]. However, cases in which symptoms developed earlier and required hospitalization have been reported, indicating that the condition may progress more rapidly and severely [7]. In this case, severe airway stenosis developed within five hours after the injury, necessitating tracheal intubation and three days of respiratory management. However, owing to appropriate airway management and comprehensive treatment, the patient is currently not experiencing any long-term complications. For pediatric airway burns, it is crucial to collaborate closely with an otolaryngologist for laryngeal observation and assessment, a pediatrician for overall management, and an anesthesiologist for airway management, from diagnosis to treatment.

Reports have not clearly demonstrated the effectiveness of antibiotics and steroids for the treatment of thermal burns of the larynx. However, in most previously reported cases of laryngeal burns, patients were treated with both steroids and antibiotics [7]. Although there is no definitive evidence, this approach is based on empirical treatment aimed at preventing airway edema progression and infection.

It is also important for parents to understand the precautions against injuries and instructions for handling emergency situations, both to prevent accidents involving their children and to educate them on avoiding serious symptoms, as seen in this case [16].

## Conclusions

In conclusion, we encountered a rare case of airway burns caused by the ingestion of hot potatoes that required tracheal intubation. This case progressed more rapidly than typical thermal burns of the larynx; however, early diagnosis and treatment were achieved through close collaboration among otolaryngologists, pediatricians, and anesthesiologists, resulting in favorable outcomes. When stridor occurs after eating hot food, thermal burns of the larynx should be considered in the differential diagnosis because prompt recognition can prevent further airway compromise. Additionally, this case underscores the need for increased awareness regarding thermal burns of the larynx caused by food consumption, particularly in young children, who may not fully comprehend the risks of consuming overly hot foods.
